# ConservedPrimers 2.0: A high-throughput pipeline for comparative genome referenced intron-flanking PCR primer design and its application in wheat SNP discovery

**DOI:** 10.1186/1471-2105-10-331

**Published:** 2009-10-13

**Authors:** Frank M You, Naxin Huo, Yong Q Gu, Gerard R Lazo, Jan Dvorak, Olin D Anderson

**Affiliations:** 1 Department of Plant Sciences, University of California, Davis, CA 95616, USA; 2 Genomics and Gene Discovery Research Unit, USDA-ARS, Western Regional Research Center, Albany, CA 94710, USA

## Abstract

**Background:**

In some genomic applications it is necessary to design large numbers of PCR primers in exons flanking one or several introns on the basis of orthologous gene sequences in related species. The primer pairs designed by this target gene approach are called "intron-flanking primers" or because they are located in exonic sequences which are usually conserved between related species, "conserved primers". They are useful for large-scale single nucleotide polymorphism (SNP) discovery and marker development, especially in species, such as wheat, for which a large number of ESTs are available but for which genome sequences and intron/exon boundaries are not available. To date, no suitable high-throughput tool is available for this purpose.

**Results:**

We have developed, the ConservedPrimers 2.0 pipeline, for designing intron-flanking primers for large-scale SNP discovery and marker development, and demonstrated its utility in wheat. This tool uses non-redundant wheat EST sequences, such as wheat contigs and singleton ESTs, and related genomic sequences, such as those of rice, as inputs. It aligns the ESTs to the genomic sequences to identify unique colinear exon blocks and predicts intron lengths. Intron-flanking primers are then designed based on the intron/exon information using the Primer3 core program or BatchPrimer3. Finally, a tab-delimited file containing intron-flanking primer pair sequences and their primer properties is generated for primer ordering and their PCR applications. Using this tool, 1,922 bin-mapped wheat ESTs (31.8% of the 6,045 in total) were found to have unique colinear exon blocks suitable for primer design and 1,821 primer pairs were designed from these single- or low-copy genes for PCR amplification and SNP discovery. With these primers and subsequently designed genome-specific primers, a total of 1,527 loci were found to contain one or more genome-specific SNPs.

**Conclusion:**

The ConservedPrimers 2.0 pipeline for designing intron-flanking primers was developed and its utility demonstrated. The tool can be used for SNP discovery, genetic variation assays and marker development for any target genome that has abundant ESTs and a related reference genome that has been fully sequenced. The ConservedPrimers 2.0 pipeline has been implemented as a command-line tool as well as a web application. Both versions are freely available at .

## Background

Single nucleotide polymorphisms (SNPs) are a valuable marker system with many genetic and genomic applications. For large scale SNP discovery, several strategies have been developed: (1) comparing sequences of multiple genotypes in the public databases to identify putative polymorphic sites [1], (2) sequencing random DNA fragments [2-4], (3) re-sequencing [5], and (4) using a target gene approach [6]. With the target gene approach, primer pairs are designed on the basis of comparisons of conserved regions (exons) of orthologous genes in related species [7]. These primers are used to amplify sequences flanking less conserved regions (such as introns) in a targeted genome. Such gene-specific PCR primer pairs can help to identify unique loci in virtually any genome. For comparative gene mapping, such loci are equivalent to sequence tagged sites (STS) or expressed sequence tags (EST). Therefore, they have been termed comparative anchor tagged sequences (CATS) [8]. Since primer pairs spanning introns are picked from two conserved exon regions of a gene, they are also called conserved primers [9], intron-flanking primers [9,10], or exon-priming-intron-crossing (EPIC) primers [8,11]. In this paper, the terms intron-flanking primers and conserved primers are used interchangeably. The general strategy of intron-flanking primer design is to align EST sequences of a targeted species with the genomic sequence of a related species for prediction of intron/exon junctions (splice sites) and estimation of intron lengths from the reference genome. The information obtained is used to design intron-flanking primer pairs for PCR amplification and sequencing from different exons through intervening introns and other exons.

The target gene approach for SNP discovery and marker development is based on three basic assumptions or findings. First, most exons or gene regions are conserved among related species [12]. Second, intron positions and approximate intron lengths of a gene are conserved features, even over long evolutionary distance [12-15]. Thus, the size of an intron between two consecutive exons can be approximately predicted from genomic sequences of related species. Third, non-coding regions (introns) of a gene evolve faster than the coding regions (exons), and are more diverse and polymorphic than the exons [16-18]. Therefore, marker development using a gene sequence with intron information is more effective. A prerequisite for this approach, satisfied now for many species, is the availability of public databases of a large number of EST sequences. By the end of 2008, a total of 59,586,036 EST entries for 1,654 species had been generated [19,20]. Most of the economically important species have large numbers of EST sequences available but lack information about their genomic sequences and intron/exon boundaries. Many of these species have a large and complex genome that cannot be sequenced efficiently and accurately with the current sequencing techniques. The target gene approach will be particularly useful for marker development in those species.

To date, the target gene approach has been used to discover SNPs and assay genetic variations in several species, such as *Rhododendron catawbiense *[10] and *Medicago truncatula *[21] using *Arabidopsis *as the reference species, and non-human vertebrates [6,8,11,22] referenced to the human or mouse genomes. However, these applications were on a small scale. No suitable high-throughput tool is available for large-scale conserved primer design. Expeditor [23] is a web-based program for designing conserved primers using human gene structure and livestock animal EST information. The major limitations of this program are its input format of reference sequences, which must be specifically formatted for the Ensembl ExonView, and limited primer design throughput due to its web implementation. GeMprospector [24,25] is another web-based pipeline tool for designing cross-species intron-flanking primers in the legume and grass families. However, this program requires ESTs of several related species in order to find conserved regions. For example, the ESTs of rice, sorghum and barley were used in an application in the grass family. Because the target sequence has to be conserved among the three species, a limited number of conserved primers can be designed. Clearly, this tool may not be suitable for the high-throughput design of conserved primers on the basis of only two related species. UniPrime2 [26,27] is the most recently released web tool for cross-species universal primer design. This tool automatically retrieves and aligns homologous sequences from GenBank, identifies conserved regions from the alignments, and generates suitable primers. However, intron regions are not taken into consideration.

For a wheat SNP project [28], the target gene approach was adopted to discover genome-specific SNPs. Wheat (*Triticum aestivum *L., 2n = 6x = 42) is an allohexaploid with the A, B and D genomes. It was formed through hybridization of three diploid species, *Triticum urartu *(AA), *Aegilops tauschii *(DD), and a wild diploid species thought to be extinct and closely related to *Aegilops speltoides *(SS) [29]. The first step in the SNP discovery pipeline was to design conserved primers, which was followed by the PCR amplification of gene targets from wheat diploid and tetraploid ancestors and sequencing of the amplicons. Genome-specific primers were then designed on the basis of amplicon sequences and verification with appropriate aneuploid genetic stocks [30]. In the second step, DNA was amplified with genome-specific primers and SNPs were discovered in amplicons [7].

To effectively design a large number of conserved primers, we developed a high-throughput pipeline software tool, named ConservedPrimers 1.0, in 2003. This tool has been further improved for general use and used in several projects for SNP discovery and genetic variation studies. In this paper, we describe the development and characteristics of ConservedPrimers 2.0 as a generalized pipeline and illustrate its utility in wheat SNP discovery.

## Intron-flanking Primer Design Pipeline and its Implementation

### The pipeline and its command-line tool

**Figure 1:**
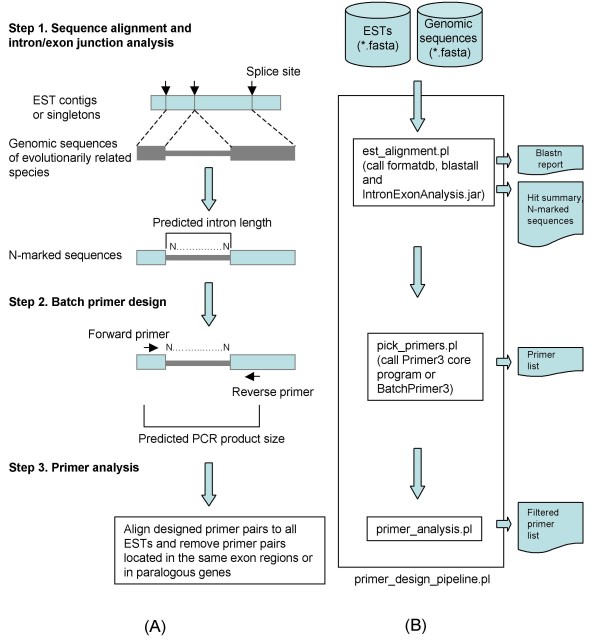
**Schematic presentation of the intron-flanking primer design pipeline, ConservedPrimers 2.0: primer design workflow (A) and command-line pipeline programs (B)**.

The pipeline (Figure 1) consists of three steps:

#### Step 1: sequence alignment and intron/exon junction analysis

**Figure 2:**
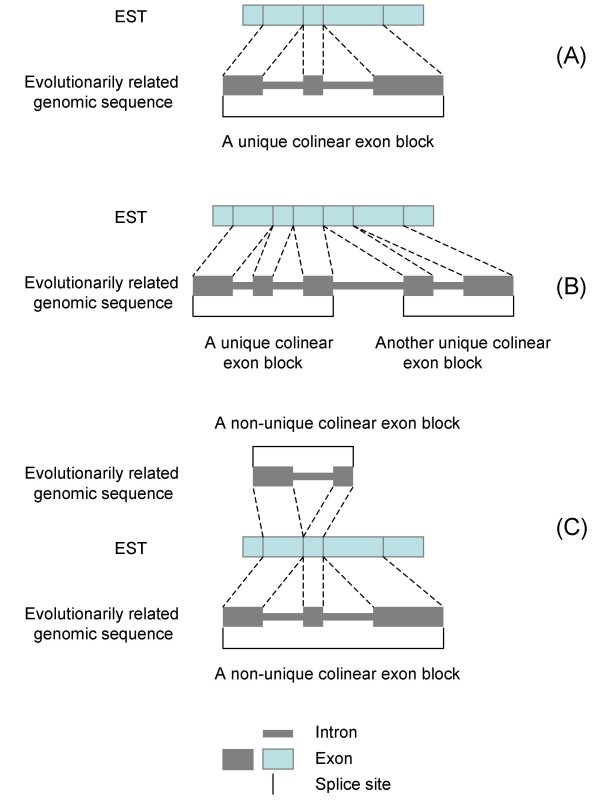
**A colinear exon block between an EST and a reference genome**. (A) A colinear exon block is found if there are two or more consecutive exon matches (i.e., one or more splice sites) within a gene and if the intron length between two consecutive exons is less than 1.5 Kb. (B) If the intron length between two consecutive exons is larger than 1.5 Kb, the alignment may be split into two smaller colinear exon blocks if possible. (C) A non-unique colinear exon block is found if the same region of an EST sequence has two or more colinear exon blocks found in different locations of the reference genome. The ESTs with non-unique colinear exon blocks are excluded from conserved primer design to avoid amplifying paralogous genes.

At this step, non-redundant ESTs of a target genome are aligned to a genomic sequence of a related species (reference genome), such as rice, *Arabidopsis*, *Brachypodium*, or human. Hereafter the term "EST" represents a singleton EST as well as an assembled EST contig which is a gene transcript assembled from a set of ESTs that appear to come from the same or closely related orthologous and paralogous genes. The use of non-redundant ESTs is strongly recommended for two reasons; i.e., to avoid duplicate primer pairs being designed for the same locus, and to facilitate identification of primers that would recognize paralogous genes. The unique EST contig sequences of a large number of species can be downloaded from the NCBI UniGene database [31] and the TIGR plant transcript assemblies database [32]. Alignments are performed with the BLASTN program (NCBI Blast 2.0) [33] to determine the existence of a colinear "exon block" between an EST and the reference genome. A colinear exon block is an intragenic linear alignment of exons along a reference genome that has at least two consecutive exon matches in the reference genome (Figure 2A). Each block must start and end with an exon and have one or more introns of less than 1.5 Kb in length. We restrict the intron length to no more than 1.5 Kb to make PCR amplification and sequencing more efficient. If an intron is longer than 1.5 Kb, the alignment should be split into two smaller colinear exon blocks if possible (Figure 2B). A single candidate primer pair should be generated from each colinear exon block, and only a single primer pair should be chosen for each gene locus. If the same region of an EST sequence has two or more colinear exon blocks detected in more than one location in the reference genome, these colinear exon blocks are considered to be non-unique (Figure 2C) and will be eliminated from further consideration. The non-unique colinear exon blocks are probably due to paralogous genes within gene families (paralogs). Paralogs originate by gene duplications either prior to speciation (outparalogs) or after speciation (inparalogs) [34]. The purpose of this step is to find a unique colinear exon block for each EST.

In order to identify colinear exon blocks and predict intron lengths from alignments of each EST sequence, BLASTN reports are further parsed and analyzed. First, one should thoroughly search for matches (alignment of an exon in an EST with the reference genome sequence) with expect values ≤ a user specified cutoff (e.g. 1e^-10^) and then identify colinear exon blocks. ESTs with non-unique colinear exon blocks are eliminated to avoid paralogous genes. ESTs with a single exon match from the BLASTN alignments will also be excluded. The length of each exon and intron will be calculated, and their coordinates determined. Finally, a new sequence string will be recorded for each EST that has a unique colinear exon block. In the sequence string, the intron sequences from the reference genome are inserted between two consecutive exons with intron sequences replaced by "Ns" (e.g., "....GATCGGTTTAC***N....N***GGTTCAATT....") - these N-marked sequences will be used to design conserved primers.

Using the N-marked sequences instead of the original EST sequences has several advantages. First, primer pairs can be more easily designed from the exons. In addition, the size of a PCR product and the number and length of introns and exons in the amplified PCR product can be estimated. Finally, in the primer analysis step (Step 3), one can check whether the designed primer pairs come from different exons or from the same exon. This process has been implemented in a Java program named "IntronExonAnalysis.jar". The entire process has been implemented in a Perl script "est_alignment.pl". This script takes two files as inputs: a FASTA file of ESTs (e.g. wheat) and a FASTA file of reference genome sequences (e.g. rice). The script first calls the formatdb program to make a BLAST database for the reference genome and then calls the blastall program (NCBI blast 2.0) [33] to perform alignments (BLASTN searches). The Java program "IntronExonAnalysis.jar" is used to parse and annotate the alignment results. This script generates two files: an intron-marked sequence file for primer design and an alignment summary file for primer analysis.

Among all of the parameters that are set in the BLASTN search, the most important parameter is the expect value (or E-value). The selection of E-value relies on the evolutionary distance between the target species and genomic reference species. For closely related species, a more stringent (smaller) value may be required. The pipeline uses 1e^-10 ^as the default.

#### Step 2: Batch primer design

Within this step, primers will be batch-designed using a Perl script (pick_primers.pl). This script takes the intron-marked sequence file exported from Step 2 as input and calls the Primer3 core program [35]. Alternatively, the BatchPrimer3 web software [9] can be used to design primers. However, some of the primer-designing parameters need to be set before running the software. The default primer-designing parameters used in the pipeline are as follows: primer length of 18 to 25 bases with the optimum 20 bases, T_m _of 55 to 65°C with the optimum 60°C, GC content of 20% to 80%, and an 800-base optimum product size with a range from 400 to 1,500 bases. Usually only one primer pair is picked for each EST. Two or more primer pair candidates may be obtained if multiple unique colinear exon blocks are found in an EST. A tab-delimited primer table file is subsequently generated from this script or from the BatchPrimer3 web software [9].

#### Step 3: Primer analysis

To increase the success rate in PCR amplification with the primers and polymorphism discovery rate, the designed primers should be unique and span at least one intron. A Perl script (primer_analysis.pl) has been implemented to analyze the designed primer pairs. This script takes several files exported from Step 1 and Step 2 and performs two major tasks. First, all designed primers are compared with all of non-redundant ESTs or other user-specified non-redundant EST databases with the BLASTN program to exclude the primer pairs with more than one hit. This step reduces failed PCR amplifications and avoids amplifying duplicate genes. The primer pairs picked from the same exon regions are also removed, since no intron can be amplified if a primer pair comes from the same exon region. Such primer pairs are removed since exon sequences are expected to have fewer polymorphisms. Information about the alignments and PCR primers from Step 1 and Step 2, such as the number of matched exons, coordinates of the exons in sequences, match scores of each exon, the numbers and lengths of introns and exons included in the amplified product, is combined in a final table of primers for primer selection and ordering.

These three steps can be done separately. However, for ease of use we have integrated them into a simple, automatic pipeline program (primer_design_pipeline.pl) that only takes two sequence files as inputs (Figure 1B). All executables and source code of the pipeline and a user's guide are available for public use [36] [see Additional file 1].

### Web implementation of the pipeline

**Figure 3:**
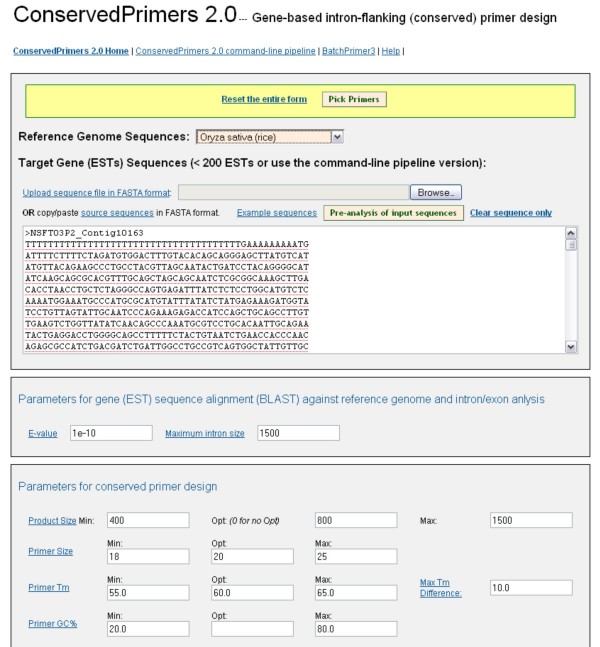
**Screenshot of the web implementation of the ConservedPrimers 2.0 pipeline**.

To assist users who need to design a small number of primers at a time, the web version of ConservedPrimers 2.0 pipeline has been implemented. A similar interface and implementations with BatchPrimer3 [9] were applied to provide users with target EST sequence input, reference sequence selection, and flexible parameter settings for BLAST search and primer design (Figure 3). Genome sequences of eight plant species, *Arabidopsis thaliana *[37], *Oryza sativa *(rice) [38], *Sorghum bicolor *(sorghum) [12,39], *Brachypodium distachyon *[40], *Glycine max *(soybean) [41], *Medicago truncatula *[42], *Vitis vinifera *(grapevine) [43] and *Populus trichocarpa *(poplar) [44], are currently available as reference sequences. More reference genome sequences can be easily embedded into the web application if needed. As in BatchPrimer3 [9], target gene (or EST) sequences can be input by either a copy-and-paste to the sequence text box (Figure 3) or uploading a sequence file. The sequences must be in FASTA format. Unlike BatchPrimer3, sequences masking with "{}", "<>" or "[]" pairs to represent including, excluding or target regions are not allowed in the sequence input. All unrecognized characters in the sequences are automatically removed. In order to balance the workload on the server, a limit of 200 sequences is set on the web application. For a larger number of EST sequences, the command-line based pipeline is recommended.

The ConservedPrimers 2.0 web application is configured to run on the Apache HTTP server [45]. It generates output in three parts: (1) a main HTML page containing the primer design summary of all input sequences, (2) an HTML table page and a tab-delimited text file listing all designed primers and primer properties, and (3) a detailed primer view page for each sequence with successfully designed primers. The primer list can be directly saved as a text file or an Excel file for further editing or primer ordering. All primer design results and intermediate files can be downloaded as a "zip" compressed file. This ConservedPrimers 2.0 web application provides a convenient and user-friendly interface and is accessible at [36].

## Results

### Intragenic colinear exon blocks between wheat ESTs and the rice genome

The command-line based pipeline was used to analyze wheat EST data. A total of 6,045 wheat ESTs (including contigs and singletons) [46] that had been mapped in wheat deletion bins were compared using the BLASTN program (NCBI blast 2.0) against the rice genome (IRGSP pseudomolecules Build04 [38]) at the expect value < 1e^-10^. A total of 4,003 ESTs (66.2%) were matched to the rice genome. Out of the 4,003 ESTs, 1,922 (58.0%) had unique colinear exon blocks, 794 (19.8%) had non-unique colinear exon blocks, and 1,287 (32.2%) had only one exon matched. The ESTs with unique colinear exon blocks were included in the pipeline for designing conserved primers.

**Figure 4:**
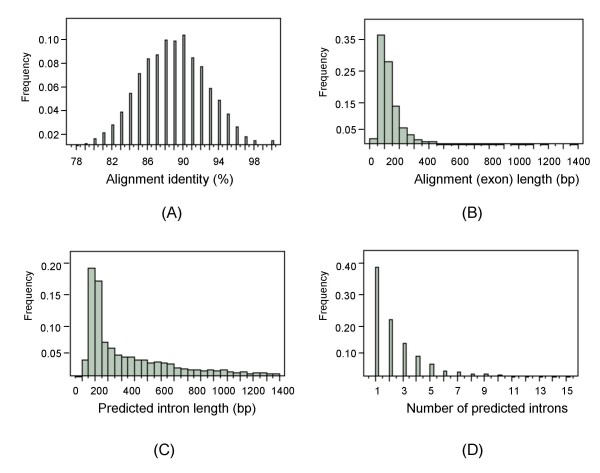
**Histograms of alignment identity (a percentage of identical matches over alignment length) (A), alignment length (B), predicted intron length (C) and number of predicted introns (D) in alignments of 1,922 wheat bin-mapped ESTs showing unique colinear exon blocks with the rice genome**. Histograms were drawn using the JMP 7.0 software (SAS Institute Inc.).

The alignment identities (percentages of identical matches over alignment lengths) over all unique colinear exon blocks were normally distributed with a mean of 88.8 ± 3.7% (Figure 4A). The alignment length or the exon length and the predicted intron length had a skewed distribution with averages of 137.2 ± 96.2 bp and 354.7 ± 341.9 bp, respectively (Figure 4B and 4C). The average number of introns in unique colinear exon blocks was 2.5 ± 2.1 (ranging from 1 to 15).

### Intron-flanking primer design, PCR amplification and SNP discovery

After removing the ESTs that had no colinear exon blocks or had non-unique colinear exon blocks, a total of 1,922 ESTs which yielded 1,975 N-marked sequences were carried forward in the pipeline for primer design. From 1,870 EST loci, 1,946 conserved primer pairs were generated with a 97% success rate for primer design. The failure in primer design for some ESTs was primarily because their N-marked sequences were too short to meet the minimum PCR product size requirement. Since the intron length was restricted to 1.5 Kb, longer colinear exon alignments were split into two or more shorter colinear exon blocks, and one N-marked sequence for each of the shorter blocks was generated for primer design. Fifty-two wheat ESTs were found to have multiple colinear exon blocks. The primers were manually screened to determine how many were derived from single- or low-copy genes in wheat. A total of 1,821 such primer pairs were used for PCR amplification and amplicon sequencing of six wheat diploid ancestors and one tetraploid wheat in a cooperative effort from seven different laboratories [47]. For example, of the 155 conserved primer pairs used for SNP discovery in wheat chromosome 1A, 1B and 1D, 145 (93.5%) produced amplicons that resulted in successful sequencing of wheat diploid ancestors and discovery of polymorphisms in at least one of the three wheat genomes.

**Figure 5:**
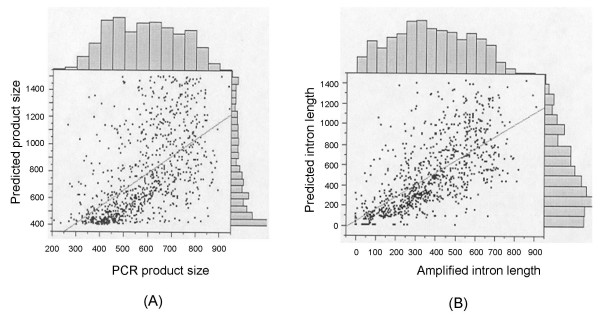
**Comparison between the total amplicon sequence sizes (bp) in wheat and the predicted PCR product sizes on the basis of rice genome sequence (A), and comparison between the amplicon intron length (bp) and the predicted intron length (bp) (B) based on alignments of wheat ESTs to the rice genome**. A total of 888 data points from 145 homologous group 1 primer pairs and their amplicons and genomic sequences were used to draw scatter plots and histograms using the JMP 7.0 software (SAS Institute Inc.). A significant regression line was fit between the total amplicon sequence sizes in wheat and the predicted PCR product sizes (A) as well as between the amplicon intron lengths and the predicted intron lengths (B). The amplified intron lengths were determined using BLAST searches of amplified genomic sequences against their corresponding ESTs.

Wheat intron lengths were correlated significantly (r^2 ^= 0.45, p < 0.0001) with the predicted intron lengths from the rice genome (Figure 5B). The same linear correlation was also observed between the actual PCR product sizes and their predicted sizes (r^2 ^= 0.33, p < 0.0001) (Figure 5A). Despite a larger genome size, the average wheat gene intron lengths were smaller (259.8 bp) than those of rice (354.7 bp) (Figure 4B). Similar results were also observed between *Gossypium*, *Arabidopsis*, and rice [14]. *Gossypium *has a genome larger than *Arabidopsis *and rice, but the *Gossypium *introns are generally smaller (149.5 bp). The difference in intron lengths between wheat and rice was small enough to allow using rice to predict wheat intron lengths in the 200 to 1,500 bp range.

Sequences of amplicons produced with the 1,821 conserved primers were used to design genome-specific primers for amplifying and sequencing the target DNA sequences from a single genome of hexaploid wheat. A total of 1,527 loci containing one or more genome-specific SNPs were discovered. In the previously mentioned 145 loci that had been mapped to wheat chromosomes 1A, 1B and 1D, a total of 114 loci (78.6%) were found to have at least one genome-specific SNP. Among them, 73 loci were located in the A genome, 64 loci in the B genome and 82 loci in the D genome.

### Running time of the ConservedPrimers pipeline

The three-step pipeline sequentially uses three separate command-line Perl scripts together with one Java program for intron/exon junction analysis, the NCBI Blast 2.0 software [33] for alignments, the Primer3 core program [35] or BatchPrimer3 [9], and three Perl packages for primer design (Figure 1B). A user can run scripts step by step, or run the single pipeline program, primer_design_pipeline.pl, which integrates all of the three steps into one script. The performance is primarily related to genome size of reference species, the number of ESTs, and the speed of the computer. For instance, 6,045 wheat ESTs and the rice genome were used for conserved primer design. The size of the rice genome (IRGSP pseudomolecules Build04) is 382 Mb. It took a desktop computer (Asus P6T, Intel core i7 920, 12 GB of RAM, and a Ubuntu Linux 9.04 64 bit operating system) a total of 9.10 minutes for running the entire pipeline and 8.46, 0.47 and 0.17 minutes for Step 1, 2 and 3, respectively. A majority of running time (93%) was taken by Step 1 to perform BLASTN searches and intron/exon analysis. The web version of the ConservedPrimers pipeline will take more time than the command-based pipeline since the sequences are loaded to the server before processing. Client internet speed will also affect sequence loading. Therefore performance of the web application may vary among different users. A random sample of 200 sequences chosen from 6,045 wheat ESTs were used for testing the web application through intranet set-up. A total of 25 seconds were needed to finish the entire pipeline.

## Discussion

### Intragenic colinear exon blocks and intron-flanking primer design

A basic prerequisite has to be satisfied for designing intron-flanking primers: both the positions and the lengths of the exon and the intron must be relatively conserved between the target genome and the reference genome, i.e., a colinear exon block must be identified in an EST locus. Extensive colinear exon blocks within genes were observed between wheat and rice. Since wheat and barley are more closely related to *Brachypodium *than to rice [48,49], *Brachypodium *may be a better reference species for comparative genomic researches than rice.

Intron length variation has been investigated in animals, plants (including rice, sorghum and maize) and fungi [12-15,50]. Although intron lengths in orthologous genes vary among species, the positions and approximate lengths of introns tend to be conserved [12,13,15,50]. Since intron lengths were significantly correlated between rice and wheat, the wheat intron lengths can be approximately predicted from the rice introns for the purpose of designing intron-flanking primers.

### ConservedPrimers 2.0 pipeline

The ConservedPrimers 2.0 pipeline is a high throughput software tool for designing intron-flanking primers, and is usable with two evolutionarily related species. The primary requirement is the availability of abundant unique ESTs for the target species and an evolutionarily related model or reference species with a fully sequenced genome. Depending on their evolutionary distance, a different E-value cutoff should be used in a BLASTN search of colinear exon blocks. For two closely related species, such as wheat, barley, sorghum, maize or sugarcane referenced to rice or *Brachypodium*, a more stringent E-value cutoff, such as 1e^-10^, should be used. Otherwise, a less stringent E-value cutoff would be more suitable. For example, *Rhododendron *is phylogenetically distant from *Arabidopsis*, and 1e^-4 ^was used in the BLAST search against *Arabidopsis *genomic sequences [10]. The more stringent the E-value cutoff, the fewer colinear exon blocks are identified. This results in fewer, but higher-quality, intron-flanking primer pairs being selected. Besides the E-value, all parameters for primer design also can be easily changed within the Perl script, pick_primers.pl.

The ConservedPrimer 2.0 pipeline has been implemented as a command-line tool as well as a web application. The command line style makes it possible to design intron-flanking primer pairs or marker candidates for polymorphism discovery in a high-throughput manner and to use any genome size of the model species and any number of the ESTs as inputs without memory and speed restrictions. The web-based pipeline serves as a convenient and easy-to-use tool for primer design of a small number of ESTs. Compared to the web-based system, a novice user may need some basic computer skills for initial set-up and running the command line programs.

## Conclusion

The ConservedPrimers 2.0 pipeline for designing intron-flanking primers was developed and its utility demonstrated. The tool can be used for SNP discovery, genetic variation assays and marker development for any target genome that has abundant ESTs and an evolutionarily related reference genome that has been fully sequenced. The ConservedPrimers 2.0 pipeline has been implemented as a command-line tool as well as a web application. Both versions are freely available at [36].

## Availability and requirements

**Project name: **ConservedPrimers pipeline software.

**Project home page: **

**Operating systems: **The command-line pipeline and the web server were tested in Linux systems, but should work in an operating system which can run Perl and Java programs.

**Programming language: **Perl and Java

**Other requirements: **Perl interpreter program, Java J2SE 1.4 or above, Primer3 core program  or BatchPrimer3 .

**License: **GNU PGL

**Any restrictions to use by non-academics: **None

## Authors' contributions

FMY designed and implemented the pipeline software, designed wheat conserved primers and drafted the manuscript. YQG and NH validated wheat conserved primers. JD, ODA and GRL helped to design the pipeline and to draft the manuscript. All authors read and approved the final manuscript.

## Supplementary Material

Additional file 1 **Conserved Primers 2.0 command-line pipeline package**. The file has been collected with "tar" and compressed by "gzip", and includes ConservedPrimers 2.0 command-line pipeline programs with source code, and a user's guide for installation. Click here for file
