# Supplementary material for: ConservedPrimers 2.0: A high-throughput pipeline for comparative genome referenced intron-flanking PCR primer design and its application in wheat SNP discovery
Source: BMC Bioinformatics. 2009 Oct 13;10:331. doi: 10.1186/1471-2105-10-331 (PMC2765976; doi:10.1186/1471-2105-10-331)
Supplement: Additional file 1 — Conserved Primers 2.0 command-line pipeline package. The file has been collected with "tar" and compressed by "gzip", and includes ConservedPrimers 2.0 command-line pipeline programs with source code, and a user's guide for installation. [file 1471-2105-10-331-S1.GZ › conserved_primers/user_guide.pdf]

The ConservedPrimers 2.0 command-line pipeline software
User's guide

Frank M. You
University of California, Davis/Genome and Gene Discovery Unit, USDA-ARS, Albany, CA
Date: January, 2009

Introduction

In order to amplify less conserved regions such as introns, primer pairs are designed in conserved regions (exons) of orthologous gene sequences from evolutionarily related species. These primers are called conserved primers, or intron-flanking primers, or exon priming intron crossing (EPIC) primers. These gene-specific PCR primer pairs may identify unique loci in virtually any plant or animal genome. Thus they have been termed comparative anchor tagged sequences (CATS).

The general strategy of intron-flanking primer design is to align EST sequences of the target genome to genomic sequences of an evolutionarily related reference genome to predict intron/exon junctions (splice sites) and to estimate intron sizes from the reference genome, which are used to design intron-flanking primer pairs for PCR amplification and sequencing of introns and the nested portions of exons.

The ConservedPrimers 2.0 command-line pipeline is a high-throughput software tool for intron-flanking primer design for SNP discovery and genetic variation assay in any target genome. The target genome needs to have abundant EST unigenes and an evolutionarily related reference genome which have been fully sequenced.

The ConservedPrimers 2.0 command-line pipeline

The pipeline consists of three separate command-line Perl scripts together with one Java program for intron/exon junction analysis, the NCBI Blast 2.0 software for alignments, the Primer3 core program or BatchPrimer3 and three Perl packages for primer design (See the section of **The software package**).

Step 1: sequence alignment and intron/exon junction analysis. At this step, alignments are performed for non-redundant EST unigenes of a target genome against the

genomic sequences of evolutionarily related species or the reference genome, such as rice, *Arabidopsis*, *Brachypodium* and human. This step was implemented in a Perl script "est_alignment.pl". This script takes two files as inputs: a FASTA file of EST unigenes and a FASTA file of reference genome sequences. The script first calls the NCBI formatdb and blastall programs (NCBI blast 2.0) to make a blast database for the reference genome and then to perform alignments (BLASTN searches), and then runs the Java program "IntronExonAnalysis.jar" to parse and annotate the alignment results. Two result files are generated from this script, including an intron-marked sequence file for primer design and an alignment summary file for primer analysis.

Step 2: Batch primer design. Within this step, primers will be batch designed using a Perl script (pick_primers.pl) which takes the intron-marked sequence file exported from Step 2 as input and calls the Primer3 core program. Alternatively, the BatchPrimer3 web software can be used to design primers. Some of primer design parameters need to be set before running the software. The default primer design parameters used in the pipeline are as follows: primer length of 18 to 25 bases with the optimum 20 bases, T_m of 55 to 65 °C with the optimum 60 °C, GC content of 20% to 80%, and the optimum product size of 800 bases ranging from 400 to 1,500 bases.

Step 3: Primer analysis. To increase the PCR amplification success rate of primers and the possibility of polymorphism discovery, a Perl script (primer_analysis.pl) was implemented to analyze the designed primer pairs. This step is to compare designed primers with all of non-redundant EST unigenes or other user-specified non-redundant EST unigene databases and to remove the primer pairs with more than one hit. This would be able to reduce the rate of failed PCR amplifications and to avoid amplifying duplicate genes. The primer pairs picked from the same exon regions are also removed. Additionally, the alignment information and PCR primers from Step 1 and Step 2, such as number of matched exons, coordinates of the exons in sequences, match scores of each exon, number and length of introns and exons included in the amplified product, are combined to generate a final primer table for further primer selection and ordering.

The three steps above can be done out separately. However, all steps have been integrated to form a simple, automatic pipeline program (primer_design_pipeline.pl) for easy use.

The command-line software package

The software package includes the following files:

(1) primer_design_pipeline.pl
(2) est_alignment.pl
(3) IntronExonAnalysis.jar
(4) pick_primers.pl
(5) primer_analysis.pl

The following three files are Perl packages for primer design using Primer3 core program.

(6) Primer.pm

(7) Primer3Output.pm

(8) PrimerPair.pm

(9) primer3_core: this is an executable binary file for primer design on the Linux operating system. The included file might not work in your computer because the "primer3_core" is a platform-dependent executable file. You should download the Primer3 source code from http://primer3.wiki.sourceforge.net/?title=Primer3_Wiki&printable=yes and then compile the source code under your own operating system and get the executable binary file "primer3_core".

In addition, two test data files are included in the package for software test.

(1) test.fasta: the target unigene file which includes 11 wheat unigenes.

(2) rice_ref.fasta: a part of rice genome sequence (chromosome 1 only).

All the files are packed in a file "conserved_primers.tar.gz".

Another third party software package, NCBI Blast software, is required for this pipeline. You need to download it from http://www.ncbi.nlm.nih.gov/BLAST/download.shtml.

Installation

1. Unpack the pipeline software package using the following command line and a directory named "conserved_primers" containing the above 11 files will be generated:

```
gunzip conserved_primers.tar.gz
tar –xvf  conserved_primers.tar
```

2. Download the Primer3 source code from http://primer3.wiki.sourceforge.net/?title=Primer3_Wiki&printable=yes and then compile the source code under your own operating system. Copy the compiled, executable file "primer3_core" to the directory "conserved_primers". If your operating system is Linux, the "primer3_core" program may be working and you don't need to download the source code again. You need to test it to see if it is working.

3. If you have no NCBI Blast software package installed in your machine, please
4. download the NCBI Blast software package from http://www.ncbi.nlm.nih.gov/BLAST/download.shtml and install the software. After installation, please set correct path pointing to the "bin" directory of the

BLAST software. Now you should have two executable files, *formatdb* and *blastall* in the bin directory which will be used in the pipeline programs.

For example, on a Linux system, you may add the following lines to the ".bashrc" file:

```
PATH=$PATH:/usr/local/blast2.0/bin
export PATH
```

Here assuming that the NCBI Blast software is installed in the */usr/local/blast2.0/*. Then type the following command line to activate the settings:

```
source .bashrc
```

5. If the path of the NCBI Blast software package is correct, you don't need to change the source code in the scripts *est_alignment.pl* and *primer_analysis.pl*. Otherwise, you need to specify the absolute paths of two programs *formatdb* and *blastall*.

Pipeline input

Two sequence files (FASTA format) are required. One is the non-redundant target unigene sequences. If you use redundant EST sequences, it is better to remove the redundant EST sequences before running the pipeline, or separately run Step 1 script "est_alignment.pl" and Step 2 script "pick_primers.pl". Because of gene redundancy in EST sequences, Step 3, Step 3, primer analysis, can not be applied. Otherwise, some useful primer pairs may be incorrectly removed. Manual checking of the primers are required.The second sequence file is the reference genome sequences.

Usage of the pipeline programs

For easy use, we give an example to show how to use the single pipeline program and how to use separate scripts step by step. The sample files are the target unigene file "test.fasta" and the reference sequence file "rice_ref.fasta" included in the software package.

1. ***Parameter setting***

In the pipeline script "primer_design_pipeline.pl", you may change the file pathe of two BLAST programs, "formatdb" and "blastall" IF you have not set up the default paths of NABI Blast software.

```
   # BLAST program. You may add the absolute path to the BLAST
programs. Assuming
```

```
# a user has correctly set the default path of the BLAST
programs.
my $blast_program = "blastall";
my $formatdb_program = "formatdb";
```

In the "est_alignment.pl", you need to set the expect value (E-value) for BLASTN search if necessary. The default value is 1e-10.

```
# Parameters for blast search
  my $e_value = '1e-10';
```

2. ***Using the single pipeline program: primer_design_pipeline.pl***

```
Usage:
perl primer_design_pipeline.pl
 -r reference genome sequence file name (fasta format)
 -i target unigene sequence file for primer design (fasta format)
 -d EST database sequence file for homology search (fasta format)
(optional)
```

For example,

```
perl primer_design_pipeline.pl –r rice_ref.fasta –i test.fasta
```

If you don't need to specify the other non-redundant EST database sequence file, the target unigene sequences will be automatically used for homology search.

```
      Create blast database and perform blastn alignment ...
      perl est_alignment.pl -b blastall -f formatdb -r rice_ref.fasta -
i test.fasta -o test.fasta_report.txt

      Performing blast againt database sequences rice_ref.fasta...
      blastall -p blastn -d rice_ref.fasta -i test.fasta -o
test.fasta_report.txt -b 10 -e 1e-10

      Intron-exon annotation and create hit file and N-marked sequences
for conserved primer design ...
       java -jar IntronExonAnalysis.jar test.fasta_report.txt
test.fasta_hits.txt 1500
      Total query sequences = 11
      Total sequence with hits = 19
      Sequences with non-unique colinear exon blocks: 1
      Sequences with unique colinear exon blocks : 9
      Total time used in Step 1: 0.0833333333333333 minutes

      Batch primer design using Primer3 core program ...
      perl pick_primers.pl -i
test.fasta_hits.txt_seqs.fasta_cleaned.fasta -o
test.fasta_primer_report.txt
      Total sequences: 11
      Total sequences with successful primers: 11
      Total time used in Step 2: 0.0166666666666667 minutes

      Primer analysis...
```

```
perl primer_analysis.pl -b blastall -f formatdb -d test.fasta -i test.fasta_primer_report.txt_seq.fasta -j test.fasta_primer_report.txt -s test.fasta_hits.txt_summary.txt_cleaned.txt

Create blast database for database sequence test.fasta ...
formatdb -p F -i test.fasta


Performing blast againt database sequences test.fasta...
blastall -p blastn -d test.fasta -i test.fasta_primer_report.txt_seq.fasta -o test.fasta_primer_report.txt_seq.fasta_blast_report.txt -b 10 -W 7 -e 1e-1
Total primer sets: 11
No homology hits are found in the designed primers.
No primer sets which are located on the same exons are found in the designed primers.
Total time used in Step 3: 0.0166666666666667 minutes
Total time used in whole pipeline: 0.116666666666667
```

Output files for the entire pipeline:

```
test.fasta_hits.txt_log.txt
test.fasta_report.txt
test.fasta_hits.txt_seqs.fasta
test.fasta_primer_report.txt_homology_check.txt
test.fasta_hits.txt_seqs.fasta_cleaned.fasta
test.fasta_primer_report.txt_seq.fasta
test.fasta_hits.txt_summary.txt
test.fasta_primer_report.txt_seq.fasta_blast_report.txt
test.fasta_hits.txt_summary.txt_cleaned.txt
test.fasta_primer_report.txt_seq.fasta_blast_report_table.txt
test.fasta_primer_report.txt
```
test.fasta_primer_report.txt_final.txt

3. *Running pipeline programs step by step*

1) Step 1:

```
Usage:
perl est_alignment.pl
  -b blastall program path
  -f formatdb program path
  -r reference genome sequence file (fasta format)
  -i target unigene sequence file (fasta format)
  -o blastn report file
```

For example,

```
perl est_alignment.pl –b blastall –f formatdb –r rice_ref.fasta –i test.fasta –o test.fasta_report.txt

blastall -p blastn -d rice_ref.fasta -i test.fasta -o test.fasta_report.txt -b 10 -e 1e-10
```

```
Intron-exon annotation and create hit file and N-marked sequences
for conserved primer design ...
    java -jar IntronExonAnalysis.jar test.fasta_report.txt
test.fasta_hits.txt 1500
   Total query sequences = 11
   Total sequence with hits = 19
   Sequences with non-unique colinear exon blocks: 1
   Sequences with unique colinear exon blocks: 9
   Total time used in Step 1: 0.0833333333333333 minutes
```

Output files for Step 1:

```
test.fasta_hits.txt_log.txt
test.fasta_report.txt
test.fasta_hits.txt_summary.txt
test.fasta_hits.txt_seqs.fasta
```
test.fasta_hits.txt_summary.txt_cleaned.txt*
test.fasta_hits.txt_seqs.fasta_cleaned.fasta*

The last two files will be used as inputs in Step 2 and Step 3.

2) Step 2:

```
Usage:
perl pick_primers.pl
     -i sequence file (fasta format)
     -o primer report file
```

The sequence file is a N-marked sequence file exported from Step 1.

For example,

```
perl pick_primers.pl –i
test.fasta_hits.txt_seqs.fasta_cleaned.fasta –o test_primer.txt

Total sequences: 11
Total sequences with successful primers: 11
Total time used in Step 2: 0.0333333333333333 minutes
```

Output files for Step 2:

test_primer.txt*
test_primer.txt_seq.fasta*

These two files will be used as inputs in Step 3.

3) Step 3:

```
perl primer_analysis.pl
        -b blastall program path (optional)
        -f formatdb program path (optional)
        -d unigene sequence file (fasta format)
        -i primer seq file from Step 2 (fasta format)
        -j primer table file from Step 2 (tab-delimited text
file)
```

```
        -s Blast hit summary file from Step 1 (tab-delimited text
file)
```

For example,

```
perl primer_analysis.pl -b blastall -f formatdb -d test.fasta -i
test_primer.txt_seq.fasta -j test_primer.txt -s
test.fasta_hits.txt_summary.txt_cleaned.txt

Create blast database for database sequence test.fasta ...
formatdb -p F -i test.fasta


Performing blast againt database sequences test.fasta...
blastall -p blastn -d test.fasta -i test_primer.txt_seq.fasta -o
test_primer.txt_seq.fasta_blast_report.txt -b 10 -W 7 -e 1e-1
Total primer sets: 11
No homology hits are found in the designed primers.
No primer sets which are located on the same exons are found in
the designed primers.
Total time used in Step 3: 0 minutes
```

Output files for Step 3:

test_primer.txt_homology_check.txt

test_primer.txt_seq.fasta_blast_report.txt

test_primer.txt_seq.fasta_blast_report_table.txt

test_primer.txt_final.txt (final primer pairs and their primer properties).
